# Students stress patterns in a Kenyan socio-cultural and economic context: toward a public health intervention

**DOI:** 10.1038/s41598-023-27608-1

**Published:** 2023-01-11

**Authors:** Victoria N. Mutiso, David M. Ndetei, Esther N. Muia, Christine Musyimi, Monicah Masake, Tom L. Osborn, Andre Sourander, John R. Weisz, Daniel Mamah

**Affiliations:** 1 Africa Mental Health Research and Training Foundation, Mawensi Road, Off Elgon Road, Mawensi Garden, P.O. Box 48423-00100, Nairobi, Kenya; 2 Department of Psychiatry, University of Nairobi, Nairobi, Kenya; 3 World Psychiatric Association Collaborating Centre for Research and Training, Nairobi, Kenya; 4 Department of Public and Community Health, Machakos University, Machakos, Kenya; 5 Shamiri Institute, Allston, MA USA; 6 Shamiri Institute, Nairobi, Kenya; 7 Department of Psychology, Harvard University, Cambridge, MA USA; 8 Department of Child Psychiatry, Turku University Hospital, Turku, Finland; 9 Departments of Psychiatry, Washington University Medical School, St. Louis, MO USA

**Keywords:** Psychology, Risk factors, Psychiatric disorders, Public health

## Abstract

This study aimed at determining the prevalence of stress, different types of stress, their severity and their determinants in Kenyan university, college and high school students. The following tools were administered to 9741 students: (1) Researcher-designed socio-demographic tool, (2) Psychiatric Diagnostic Screening Questionnaire (PDSQ) for psychiatric disorders, (3) WERC Stress Screen for stress, (4) Washington Early Recognition Center Affectivity and Psychosis (WERCAP) screen for psychosis and affectivity, (5) Wealth Index Questionnaire for economic indicators. Descriptive analysis for the prevalence of different types of stress and inferential analysis for stress and independent variables were done. Significant variables (*p* < 0.05) were fitted into generalized linear model to determine independent predictors. The mean age of the respondents was 21.4 years (range 16–43). Money issues were the commonest stressors while alcohol and drug use were the least. The independent predictors of stress were females, college students and use of gas stove. In conclusion, up to 30% of the students suffer from mild to severe stress. The students experience a wide range of stressors. The most important stressors include money and finances, family related problems and concerns about their future. Our findings suggest a public health approach to create stress awareness in students.

## Introduction

Stress has been variously defined as any external event or any internal drive which threatens to upset the organism’s equilibrium”^1^, and as a condition or feeling that a person experiences when they perceive that the demands exceed the personal and social resources the individual can mobilize^2^. For most people, stress is a negative experience. Excesses of social, environmental, or physical stress can have destabilizing effects on individual, physiological and general functioning^3^. Stress is a common phenomenon that cuts across race or cultural background^4^, and any developmental stage^5^. This is particularly true during the changeover from adolescence to adulthood in college students^6^. In this stage, university and college students face fast physical, social and mental changes during which they may not have the experience to adapt^7^. College students experience more multifaceted inconveniences due to academic pressure, adaptation to a new environment, fear of failure, struggle to create uniqueness, inferiority, and efforts to attain social familiarity^8^.

### Prevalence of stress and the different stressors

Stress prevalence among students varies widely from 34.5% in Spain^9^, 20.2% in China^10^ and 4.1% in Ethiopia^11^. This variation can be attributed to the methodology used in each study and the period in which the studies were conducted. However, of noteworthy is that most of the studies on student stress have been done on medical students^3,12^ with very few on non-medical students^13^. In Kenya, a study conducted on medical students found 61.6% of the students to have moderate stress^14^ while another study on stress and psychosocial adjustment among non-medical students reported that 35.6% had a low score, 27.4% moderate and 37.0% were classified as having high stress levels^13^.

Students’ stressors may include the pressure of expectation to succeed, an uncertain future, difficulties in integrating into the system, social, emotional and physical and family problems. Other stressors include the transitional nature of college and university life, feelings of loneliness, low social support, nervousness and worrying about academic results, making new friends, and adjusting to a new place away from home at a time when they do not know how to deal with these new experiences^15–19^. In addition, difficulties in time management, financial matters, interactions with lecturers, personal subjective goals, adjustment problem in the academic culture and lack of support systems can cause stress among students^20–24^.

### Stress indicators/determinants

Studies at different tiers of education and different disciplines have reported an association between socio demographic characteristics and stress. For instance, female students have been reported to have more stressors than males^3,7,11,15,25–27^. Females tend to internalize stress more than males^28^. A study in Nigeria also found age to have a significant relationship with perceived stress^15^ with a systematic review of 38 studies reporting higher level of stress in the younger students than older students^29^. However, some other studies have not found a significant association between selected personal attributes including age, gender, marital status, study level, faculty (group of university departments) among others and perceived stress^30,31^. In addition, a study on students in China on association between socioeconomic status and uncertainty stress found some of the socioeconomic status variables studied—parents occupation, type of residence, family income to be associated with stress^32^. In the Kenyan context, the following variables have been used to determine a family’s socioeconomic status: availability or non-availability of electricity, television, refrigerator and toilet; type of house floor; source of water and cooking method^33^. These variables can influence the stress level with an example of exposure to television which has been found to increase stress levels^34^. Other factors that have been found to be associated with stress in students include: smoking, alcohol consumption, insomnia, drug abuse and other psychiatric disorders such as depression^9,14,35–37^.

### The consequences of stress

These include impaired judgment, absenteeism, self-medication, and addiction to substances like khat chewing, smoking cigarettes, and drinking alcohol^20^. Chronic exposure to stressful conditions leads to deterioration of academic performance, loss of memory, poor relationships with peers and family members, and overall dissatisfaction with life^38^; impaired immune system, suppressed fertility, digestive problems, loss of appetite, increased anxiety, and depression that can ultimately lead to suicide^3,27,39,40^.

### Gaps in previous studies

1. No concurrent studies using the same instruments for different tiers of education (high school, college and university) in a way that allows comparisons between these different tiers.
2. No epidemiological patterns and prevalence of the different types of stressors in a way that informs prioritization of intervention.
3. Scanty documentation of associated factors beyond gender and therefore limited understanding of stress and its context, further limiting informed and focused intervention.

### Aims of this study

1. To use the same methods to concurrently study stressors in high school, college and university students.
2. To determine the prevalence of different types of stressors.
3. To document the various associated factors and predictors of stress: social-demographic variables; mental disorders including alcohol and substance use and wealth and economic environmental factors.
4. To use our findings to suggest an informed, integrated and context-appropriate approach to intervention.

## Methods

### Data collection and recruitment

Human subject procedures were approved by the Maseno University Ethics Review Board (IRB # MSU/DRPI/MUERC/00344/16) and the Institutional Review Board of Washington University in St. Louis. All methods were performed in accordance with the relevant guidelines and regulations of the review board.

The participants who took part in the study were all Kenyans, recruited from universities, community and mid-level colleges, and high schools as well in Nairobi and three counties in South Eastern Kenya: Machakos, Kitui and Makueni Counties. At the time of recruitment, high school students were home as the schools were closed while participants from the tertiary institutions were approached after their lecture hours. We sought the permission of institutional heads for the colleges and the universities, and community leadership for the schools. Only participants that were within the required age bracket, able to read, write and speak English since English is the language used in educational institutions in Kenya, as well as voluntarily agreed to take part in the study were included in the study by signing informed consent forms. For participants less than 18 years, consent was obtained from parents and guardians. The total number of participants included in the study was 9741.

### Instruments

1. *Socio-demographic characteristics*: A researcher-designed questionnaire was used on the respondents to get their socio-demographic information. The socio-demographic variables included gender, age, marital status, religion, birth order, number of siblings, level of education and living status.
2. *WERC Stress Screen*: It is a self-report questionnaire used to assess the total stress burden and the severity of individual stressors. It has been found to correlate well with various mental disorders^36^. There are 23 questions inquiring about the individual stressors. The effects of the stressors are measured and recorded as a Likert scale i.e. No, A little, Moderate, A lot and Severely.
3. *The Psychiatric Diagnostic Screening Questionnaire (PDSQ)*: A self-report scale consisting of 126 questions assessing symptoms of 13 DSM IV axis I disorders^41^: anxiety disorders (panic disorder, agoraphobia, PTSD, obsessive–compulsive disorder, generalized anxiety disorder [GAD], and social phobia); substance use disorders (alcohol abuse/dependence and drug abuse/dependence); and somatoform disorders (somatization disorder and hypochondriasis), with an additional 6-item psychosis screen. Suicidal ideation is measured by the last six questions on major depressive episode domain, classified as: frequently thinking of dying in passive ways like going to sleep and not waking up, wishing to be dead, thinking you were better off dead, having thoughts of suicide, seriously considering taking life, and thinking about specific ways of taking your life. The questions were coded as No or Yes with No having a value of zero and Yes having a value of one. PDSQ has good psychometric properties such that the subscales showed good, almost excellent levels of internal consistency from a validity study involving 994 psychiatric outpatients^41^. The Cronbach’s α was greater than 0.80 for 12 subscales among the 13, with the mean α coefficient being 0.86. Test–retest reliability coefficient was 0.83, examined in 185 participants who completed the PDSQ twice in less than a week. A total of 361 non-clinical participants who completed questionnaires at home for less than a week, were used to examine the discriminant and convergent validity of PDSQ^42^.
4. *The Washington Early Recognition Center Affectivity and Psychosis (WERCAP) screen*: This tool has been validated in Kenya^43^ assessing psychosis-risk symptoms and bipolar-risk symptoms (“affectivity”) based on symptom frequency and effects on functioning^36,44^, with high test–retest reliability and validity with affectivity’s sensitivity of 0.91 and specificity of 0.7,1 and psychosis sensitivity of 0.88 and specificity of 0.82.
5. *Wealth Index Questionnaire*: This tool is based on the World Bank Recommendation for LMIC^45^, which has been adopted by the Kenyan government for its use in Kenya. It has questions concerning household items, type of housing, type of toil, source of energy and source of water. The following is a description of some of the items and their significance as economic indicators in the Kenyan context. Household connection to electricity supply is still an indicator of good economic status but with the Government policy for electricity supply to every household paid for by the Government, this indicator may become less indicative of economic status with time. Graduation from availability of a radio in the house to television and a refrigerator is a reflection of economic affordability and so is graduation from a bicycle to a motorcycle to a motor vehicle. In the Kenyan context, the quality and comfort of the house in which the family live is directly related to the economic status of the family. Earth floor means that the floor of the house is the bare ground without any cover and consists only of soil because they cannot afford a better cover. A cement floor means the family can make a concrete slab to cover the floor which can be done on site and with relatively minimal cost. Tiles which are bought from manufacturers are ceramic and much more expensive to install than cement floor. A wood floor is more exotic and the most expensive. Other indicators of economic status vary with the kind of toilet the family uses ranging from no toilet (going to the bush) to a pit latrine and a flush toilet. And so are the sources of water and methods used in cooking. These questions are used to estimate the socio-economic standing by coming up with a wealth index, grouped into 5 quintiles with 5 representing the highest level and 1 lowest level.

### Statistical analysis

Analysis was done in Statistical Package for the Social Sciences (SPSS) version 23.0 after the data was coded and cleaned. Statistical and exploratory data analysis results were presented in tables. Descriptive statistics were used to estimate the various stressors, as well as include the participants' characteristics. Mean and standard deviation for the various stressors were calculated. Differences in mean scores of stressors across gender, level of education, living status and age were examined using independent t-test and one way analysis of variance (ANOVA). The total score of all the stressors was used and compared with other variables to check for the association between them. All the tests in the analysis were two-sided, with alpha set at *p* < 0.05. Univariate analysis between total stress score and categorical variables were estimated using an independent t-test and one way analysis of variance (ANOVA) in which means of the variables were compared. Correlation analysis was computed to draw inferences between total stress score and numerical variables. Variables with a *p*-value of less than 0.05 were fitted into a generalized linear model with normal distribution and identity link function to identify independent predictors of stress. The strength and significance of the association between stress and the independent predictors was assessed by beta coefficients with a 95% confidence interval. The wealth index was not included in the generalized model because of the high collinearity between the wealth index and the socio-economic indicators/factors.

### Ethics approval and consent to participate

Ethical approval for this study was obtained from Maseno University Ethics Review Committee (IRB # MSU/DRPI/MUERC/00344/16) in Kenya and the Institutional Review Board of Washington University in St. Louis. All those aged 18 + were approached for informed consent; for those under 18, informed consent was sought and obtained from their guardians and then informed assent was obtained from them in addition to the consent of their guardians.

## Results

### Response rate

All the 9,741 students who were approached to participate gave informed consent and participated in the study giving a response rate of 100%. The response rate for individual items questions varied but nearly all of them were not less than 98% including a 99.3% (9672 out 9741) response rate on gender.

### Socio-demographic and wealth index

The socio-demographic characteristics of the respondents are summarized in Table 1. There were 9741 respondents with a mean age of 21.4 years (range 16–43) of whom 53.5% were males. The majority (93.3%) were single, living with both parents (79.9%), between ages 21–24 years (51.7%) and were 1st or 2nd born in their families. The wealth index was evenly distributed with quintile 5 being the lowest (16.6%).

**Table 1:** Socio-demographic characteristics of respondents.

Variable	Category	n (%)	Stress score	*F*/t	*p*-value
			Mean ± SD		
Gender	Male	5172 (53.5)	25.1 ± 26.4	− 3.717	** < 0.001**
	Female	4500 (46.5)	27.2 ± 28.1		
Age (years)	15–17	581 (6.0)	27.7 ± 29.1	2.989	**0.030**
	18–20	3635 (37.3)	25.1 ± 26.5		
	21–24	5041 (51.7)	26.3 ± 27.4		
	≥ 25	484 (5.0)	27.6 ± 27.9		
Marital status	Married	607 (6.3)	26.8 ± 28.0	1.316	0.268
	Single	9056 (93.3)	25.9 ± 27.1		
	Others	38 (0.4)	32.3 ± 27.8		
Religion	Protestant	5512 (57.1)	25.3 ± 27.0	3.461	**0.016**
	Catholic	3358 (34.8)	26.8 ± 27.2		
	Muslim	410 (4.3)	26.8 ± 29.4		
	Other	368 (3.8)	28.8 ± 28.1		
Birth order	1–2	5538 (56.9)	25.8 ± 26.9	1.125	0.325
	3–5	3271 (33.6)	26.0 ± 27.3		
	6 +	920 (9.5)	27.2 ± 28.6		
Number of siblings	0–3	4544 (46.7)	24.6 ± 25.7	− 4.810	** < 0.001**
	≥ 4	5184 (53.3)	27.2 ± 28.4		
Level of education	High School	1506 (15.6)	28.1 ± 27.7	11.593	** < 0.001**
	College	1534 (15.8)	27.9 ± 28.2		
	University	6647 (68.6)	25.1 ± 26.8		
Living status	Both Parents	7444 (79.9)	25.8 ± 27.3	1.062	0.346
	Single Parent	1622 (17.4)	26.5 ± 27.4		
	Others	256 (2.7)	27.9 ± 26.9		
Wealth Index	Quintile 1	2043 (21.0)	28.6 ± 29.5	9.256	** < 0.001**
	Quintile 2	1865 (19.1)	27.2 ± 27.9		
	Quintile 3	2002 (20.6)	24.5 ± 26.6		
	Quintile 4	2214 (22.7)	24.9 ± 25.8		
	Quintile 5	1617 (16.6)	24.6 ± 25.6		

*F* one way anova test, *t* independent t-test, *p-value* significance level, *SD* Standard deviation.

Significant values are in bold.

### Patterns of stress

**Figure 1:**
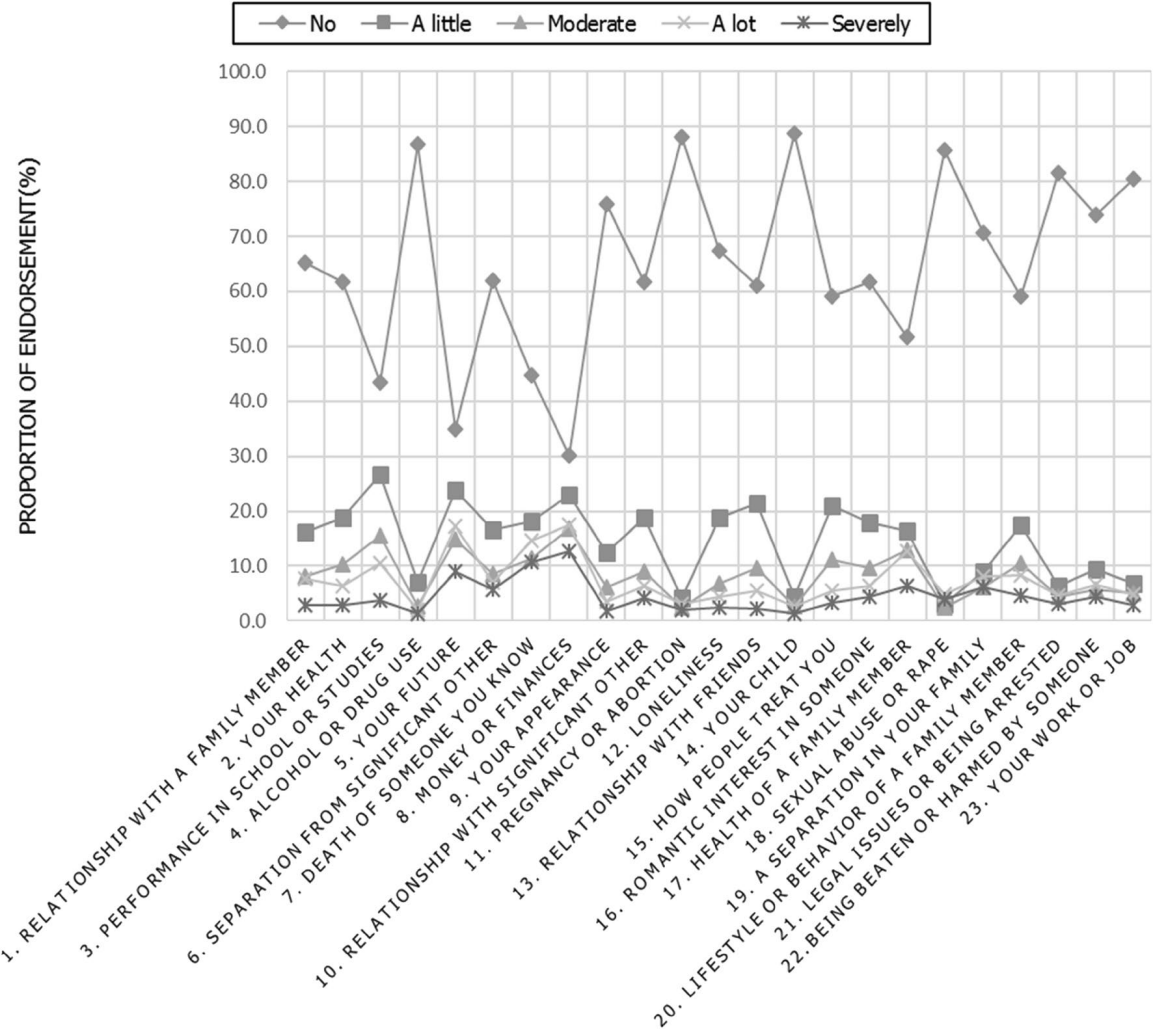
Individual endorsements to various stressors in life.

There was a wide variation in the scores of severity with the different types of stressors, with the majority scoring "no" and the least number scoring for severe as seen in Fig. 1. The leading stressors that scored "no" included alcohol and drug use, pregnancy and abortion, youth child and sexual abuse/rape. The stressors that scored least on "no" were your future, money/finances.

**Figure 2:**
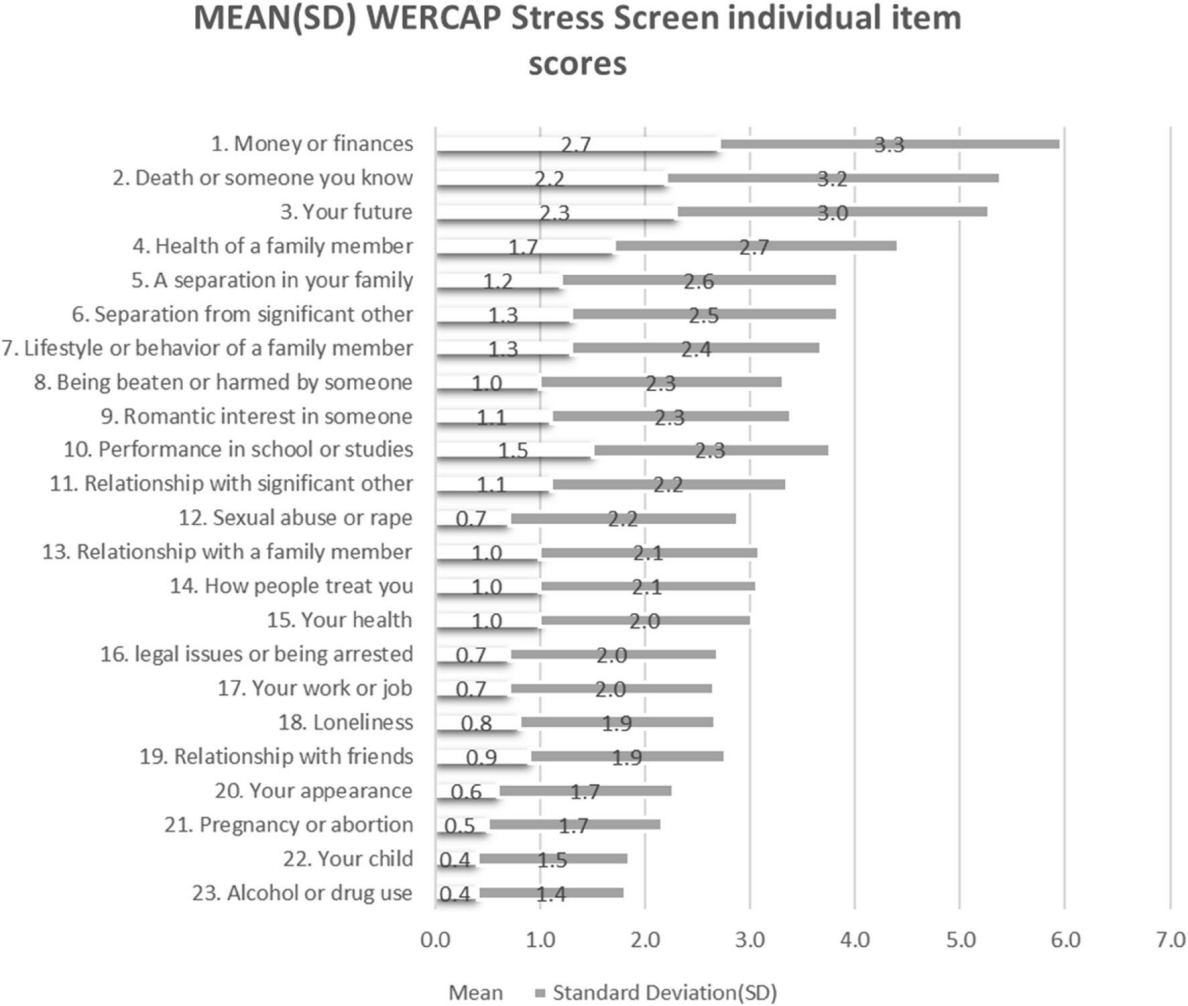
Mean and standard deviation WERCAP stress items scores.

**Figure 3:**
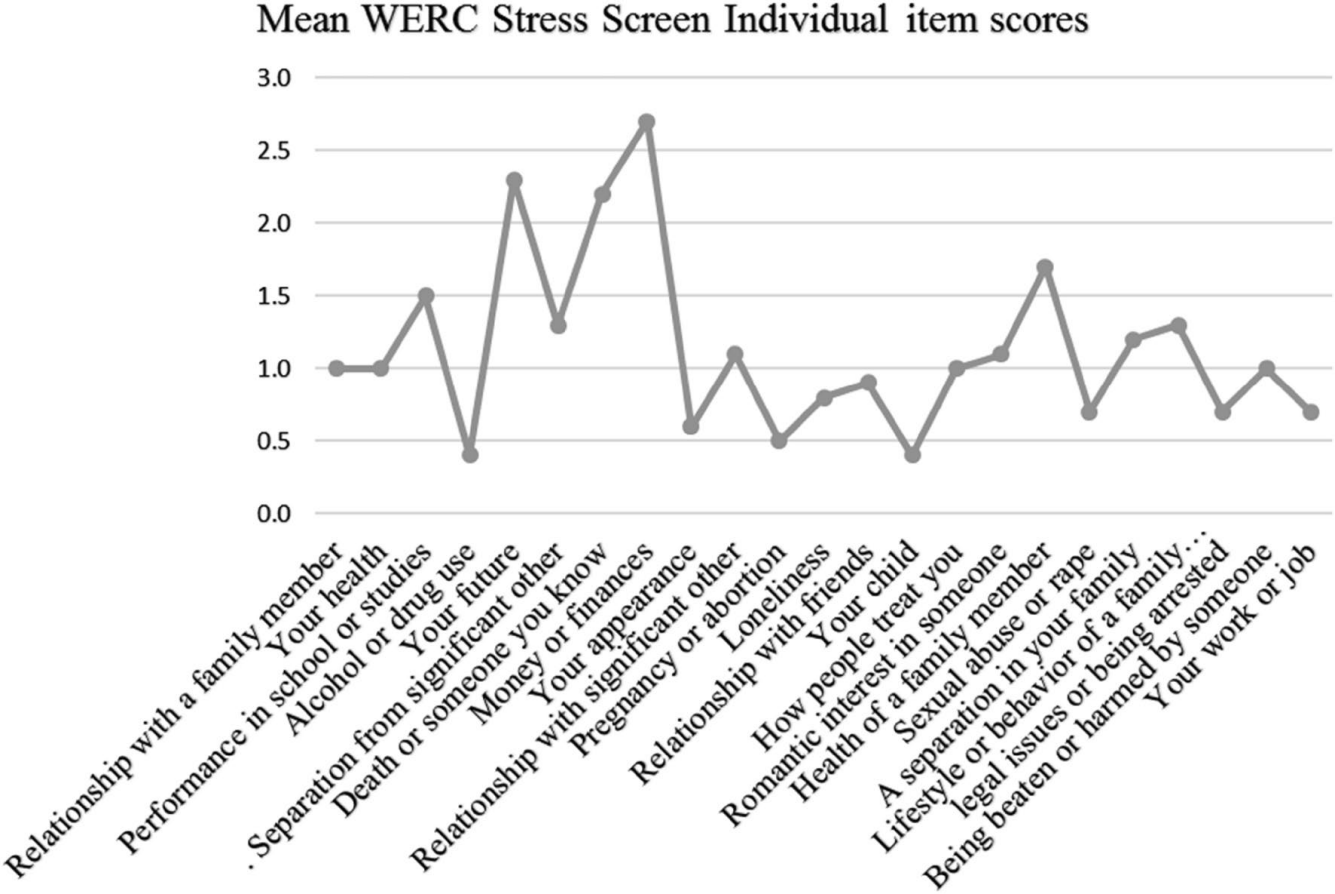
Mean WERCAP stress items scores.

Figure 2 summarizes the mean scores for the various stressors with the attendant standard deviations (descending order), while Fig. 3 summarizes the same in a quick visual graphic from the varied patterns of mean scores of the individual item score. Money/finances, worry about future, and the death of someone you know were the leading stressors. Table 2 summarizes the mean score difference in stressors among various groups i.e. gender, level of education, living status, and age. Gender had the highest number of significant differences in stressors (16 out of 23) while living status had the least number of significant differences in stressors (5 out of 23).

**Table 2:** Mean and standard deviation of WERC Stress item scores.

Variable	Category	Overall N = 9741	Gender		*p*-value^t^	Level of education			*p*-value^F^		
			Male n = 5172	Female n = 4500		High School n = 1506	College n = 1534	University n = 6647			
8. Money or finances	Mean (SD)	2.71 (3.26)	2.67 (3.25)	2.77 (3.28)	0.119	1.96 (2.98)	2.87 (3.31)	2.86 (3.29)	** < 0.001**		
5. Your future	Mean (SD)	2.31 (2.98)	2.31 (3.01)	2.30 (2.94)	0.901	2.13 (2.95)	2.42 (2.99)	2.31 (2.98)	**0.027**		
7. Death of someone you know	Mean (SD)	2.23 (3.19)	1.99 (3.01)	2.51 (3.37)	** < 0.001**	2.12 (3.08)	2.39 (3.31)	2.22 (3.19)	0.058		
17. Health of a family member	Mean (SD)	1.69 (2.71)	1.54 (2.56)	1.87 (2.86)	** < 0.001**	1.70 (2.81)	1.84 (2.86)	1.66 (2.65)	0.059		
3. Performance in school or studies	Mean (SD)	1.48 (2.26)	1.43 (2.23)	1.55 (2.29)	**0.006**	1.64 (2.48)	1.59 (2.36)	1.42 (2.17)	** < 0.001**		
6. Separation from significant other	Mean (SD)	1.26 (2.53)	1.19 (2.45)	1.35 (2.63)	**0.002**	1.24 (2.62)	1.30 (2.53)	1.26 (2.51)	0.817		
20. Lifestyle or behavior of a family member	Mean (SD)	1.26 (2.38)	1.13 (2.21)	1.41 (2.56)	** < 0.001**	1.36 (2.46)	1.40 (2.58)	1.20 (2.31)	**0.002**		
19. A separation in your family	Mean (SD)	1.23 (2.64)	1.14 (2.49)	1.34 (2.80)	** < 0.001**	1.12 (2.40)	1.36 (2.75)	1.23 (2.67)	**0.048**		
16. Romantic interest in someone	Mean (SD)	1.13 (2.29)	1.31 (2.44)	0.93 (2.09)	** < 0.001**	1.23 (2.47)	1.16 (2.39)	1.09 (2.22)	0.086		
10. Relationship with significant other	Mean (SD)	1.10 (2.25)	1.03 (2.20)	1.18 (2.31)	**0.002**	1.17 (2.47)	1.25 (2.38)	1.05 (2.16)	**0.003**		
15. How people treat you	Mean (SD)	1.04 (2.06)	1.01 (2.03)	1.07 (2.10)	0.111	1.43 (2.54)	1.14 (2.17)	0.92 (1.89)	** < 0.001**		
1. Relationship with a family member	Mean (SD)	1.00 (2.08)	0.98 (2.09)	1.03 (2.08)	0.312	1.52 (2.63)	1.02 (2.06)	0.88 (1.92)	** < 0.001**		
2. Your health	Mean (SD)	1.00 (2.01)	0.91 (1.95)	1.10 (2.09)	** < 0.001**	1.34 (2.45)	1.17 (2.19)	0.88 (1.84)	** < 0.001**		
22. Being beaten or harmed by someone	Mean (SD)	0.97 (2.32)	0.92 (2.19)	1.04 (2.47)	**0.013**	1.02 (2.30)	1.07 (2.40)	0.94 (2.31)	0.141		
13. Relationship with friends	Mean (SD)	0.91 (1.86)	0.92 (1.91)	0.91 (1.81)	0.745	1.45 (2.55)	0.95 (1.87)	0.78 (1.64)	** < 0.001**		
12. Loneliness	Mean (SD)	0.80 (1.87)	0.75 (1.81)	0.86 (1.93)	**0.005**	0.88 (2.05)	0.85 (1.92)	0.77 (1.81)	0.093		
18. Sexual abuse or rape	Mean (SD)	0.72 (2.19)	0.65 (2.03)	0.80 (2.35)	** < 0.001**	0.75 (2.11)	0.75 (2.25)	0.70 (2.19)	0.649		
23. Your work or job	Mean (SD)	0.70 (1.96)	0.74 (1.99)	0.66 (1.92)	**0.047**	0.83 (2.14)	0.80 (2.10)	0.64 (1.88)	** < 0.001**		
21. legal issues or being arrested	Mean (SD)	0.68 (1.99)	0.71 (1.96)	0.65 (2.02)	0.142	0.75 (2.00)	0.68 (1.95)	0.67 (2.00)	0.437		
9. Your appearance	Mean (SD)	0.62 (1.67)	0.61 (1.65)	0.63 (1.70)	0.475	0.90 (2.03)	0.61 (1.59)	0.56 (1.58)	** < 0.001**		
11. Pregnancy or abortion	Mean (SD)	0.45 (1.66)	0.36 (1.45)	0.56 (1.87)	** < 0.001**	0.53 (1.69)	0.45 (1.66)	0.44 (1.66)	0.162		
14. Your child	Mean (SD)	0.37 (1.45)	0.33 (1.36)	0.43 (1.56)	** < 0.001**	0.51 (1.67)	0.44 (1.58)	0.33 (1.36)	** < 0.001**		
4. Alcohol or drug use	Mean (SD)	0.37 (1.42)	0.49 (1.61)	0.24 (1.16)	** < 0.001**	0.53 (1.76)	0.40 (1.50)	0.33 (1.29)	** < 0.001**		

Variable	Category	Overall N = 9741	Living status			*p*-value^F^	Age (years)				*p*-value^F^
			Both parents n = 7444	Single parent n = 1622	No parent n = 256		15–17 n = 581	18–20 n = 3635	21–24 n = 5041	≥ 25 n = 484	
8. Money or finances	Mean (SD)	2.71 (3.26)	2.67 (3.23)	2.87 (3.37)	2.84 (3.45)	0.077	1.43 (2.69)	2.59 (3.20)	2.92 (3.32)	3.02 (3.30)	** < 0.001**
5. Your future	Mean (SD)	2.31 (2.98)	2.28 (2.96)	2.33 (3.06)	2.66 (3.17)	0.125	2.12 (3.06)	2.23 (2.92)	2.36 (3.01)	2.52 (3.00)	**0.034**
7. Death of someone you know	Mean (SD)	2.23 (3.19)	2.02 (3.05)	2.99 (3.55)	3.46 (3.79)	** < 0.001**	1.82 (2.97)	2.17 (3.12)	2.28 (3.22)	2.57 (3.55)	** < 0.001**
17. Health of a family member	Mean (SD)	1.69 (2.71)	1.67 (2.70)	1.70 (2.70)	1.94 (3.01)	0.296	1.70 (2.91)	1.65 (2.65)	1.70 (2.71)	1.81 (2.80)	0.625
3. Performance in school or studies	Mean (SD)	1.48 (2.26)	1.48 (2.24)	1.49 (2.34)	1.32 (1.92)	0.532	1.96 (2.84)	1.44 (2.27)	1.45 (2.16)	1.56 (2.28)	** < 0.001**
6. Separation from significant other	Mean (SD)	1.26 (2.53)	1.28 (2.53)	1.24 (2.55)	1.02 (2.35)	0.241	1.06 (2.40)	1.23 (2.46)	1.32 (2.60)	1.20 (2.42)	0.066
20. Lifestyle or behavior of a family member	Mean (SD)	1.26 (2.38)	1.24 (2.35)	1.31 (2.50)	1.38 (2.50)	0.389	1.33 (2.48)	1.18 (2.30)	1.30 (2.41)	1.35 (2.48)	0.089
19. A separation in your family	Mean (SD)	1.23 (2.64)	1.23 (2.64)	1.17 (2.57)	1.36 (2.79)	0.500	1.11 (2.46)	1.21 (2.63)	1.27 (2.67)	1.16 (2.57)	0.412
16. Romantic interest in someone	Mean (SD)	1.13 (2.29)	1.15 (2.30)	1.06 (2.25)	0.98 (2.25)	0.188	1.30 (2.65)	1.11 (2.25)	1.14 (2.30)	0.89 (1.97)	**0.033**
10. Relationship with significant other	Mean (SD)	1.10 (2.25)	1.13 (2.27)	1.05 (2.28)	0.78 (1.81)	**0.026**	1.15 (2.47)	1.06 (2.21)	1.12 (2.27)	0.99 (2.02)	0.383
15. How people treat you	Mean (SD)	1.04 (2.06)	1.03 (2.05)	1.03 (2.06)	1.34 (2.48)	0.061	1.43 (2.64)	1.01 (2.04)	1.00 (1.99)	1.13 (2.09)	** < 0.001**
1. Relationship with a family member	Mean (SD)	1.00 (2.08)	0.96 (2.05)	1.10 (2.15)	1.22 (2.33)	**0.012**	1.72 (2.92)	0.94 (2.00)	0.94 (1.98)	1.21 (2.33)	** < 0.001**
2. Your health	Mean (SD)	1.00 (2.01)	1.01 (2.04)	0.93 (1.91)	0.98 (2.13)	0.378	1.51 (2.68)	1.01 (2.02)	0.93 (1.91)	0.99 (1.97)	** < 0.001**
22. Being beaten or harmed by someone	Mean (SD)	0.97 (2.32)	0.98 (2.32)	0.95 (2.35)	0.95 (2.28)	0.905	1.12 (2.48)	0.93 (2.23)	1.00 (2.38)	0.88 (2.18)	0.158
13. Relationship with friends	Mean (SD)	0.91 (1.86)	0.93 (1.87)	0.80 (1.73)	1.00 (2.16)	**0.028**	1.69 (2.80)	0.93 (1.88)	0.82 (1.71)	0.82 (1.65)	** < 0.001**
12. Loneliness	Mean (SD)	0.80 (1.87)	0.79 (1.84)	0.82 (1.92)	1.09 (2.39)	**0.048**	0.98 (2.27)	0.80 (1.87)	0.79 (1.83)	0.78 (1.73)	0.141
18. Sexual abuse or rape	Mean (SD)	0.72 (2.19)	0.74 (2.22)	0.65 (2.12)	0.54 (1.85)	0.123	0.63 (1.92)	0.69 (2.19)	0.75 (2.22)	0.69 (2.08)	0.519
23. Your work or job	Mean (SD)	0.70 (1.96)	0.69 (1.94)	0.73 (2.05)	0.62 (1.67)	0.647	0.57 (1.79)	0.61 (1.88)	0.74 (1.97)	1.03 (2.47)	** < 0.001**
21. legal issues or being arrested	Mean (SD)	0.68 (1.99)	0.71 (2.01)	0.63 (1.96)	0.59 (1.76)	0.246	0.66 (2.01)	0.61 (1.85)	0.74 (2.07)	0.76 (2.08)	**0.021**
9. Your appearance	Mean (SD)	0.62 (1.67)	0.62 (1.67)	0.59 (1.64)	0.69 (1.89)	0.588	0.98 (2.14)	0.61 (1.63)	0.58 (1.61)	0.65 (1.76)	** < 0.001**
11. Pregnancy or abortion	Mean (SD)	0.45 (1.66)	0.47 (1.71)	0.42 (1.60)	0.39 (1.45)	0.344	0.59 (1.89)	0.44 (1.63)	0.45 (1.67)	0.37 (1.47)	0.146
14. Your child	Mean (SD)	0.37 (1.45)	0.37 (1.46)	0.42 (1.57)	0.31 (1.10)	0.360	0.43 (1.52)	0.29 (1.31)	0.39 (1.48)	0.75 (1.99)	** < 0.001**
4. Alcohol or drug use	Mean (SD)	0.37 (1.42)	0.39 (1.47)	0.31 (1.24)	0.43 (1.53)	0.091	0.43 (1.56)	0.34 (1.36)	0.38 (1.41)	0.52 (1.69)	**0.030**

Mean scores on a 5 point scale (0 = No, 1 = A little, 2 = Moderate, 5 = A lot, 10 = Severely); *F* one way anova test, *t* independent t-test, *p-value* significance level *SD* Standard deviation.

Significant values are in bold.

### Associations between stress and socio-demographics

These are summarized in Table 1. The following were significantly associated with stress: female gender, age, religion, high number of siblings, level of education and low level wealth index.

### Associations between socio-economic indicators and stress

These are summarized in Table 3. There were significant negative association between stress and electricity, television, cement floor, piped water and gas stove cooking method. Household economic indicators positively associated with stress were earth floor, well water, surface water, no toilet, firewood cooking method and kerosene stove cooking method.

**Table 3:** Economic characteristics of respondents.

Variable	Category	N	Frequency	Stress Score	*p*-value
				Pearson correlation coefficient, r	
Electricity	Mean (SD)	9741	0.64 ± 0.48	− 0.046	** < 0.001**
Radio	Mean (SD)	9741	0.83 ± 0.38	− 0.011	0.259
Television	Mean (SD)	9741	0.62 ± 0.49	− 0.039	** < 0.001**
Refrigerator	Mean (SD)	9741	0.24 ± 0.42	− 0.019	0.060
Cell phone	Mean (SD)	9741	0.76 ± 0.43	− 0.015	0.138
Bicycle	Mean (SD)	9741	0.39 ± 0.49	− 0.002	0.872
Motorcycle	Mean (SD)	9741	0.20 ± 0.40	− 0.010	0.335
Motor vehicle	Mean (SD)	9741	0.19 ± 0.40	− 0.018	0.071
Earth floor	Mean (SD)	9741	0.23 ± 0.42	0.070	** < 0.001**
Cement floor	Mean (SD)	9741	0.57 ± 0.50	− 0.050	** < 0.001**
Tile floor	Mean (SD)	9741	0.19 ± 0.39	− 0.019	0.055
Wood floor	Mean (SD)	9741	0.02 ± 0.14	0.018	0.079
Other floor material	Mean (SD)	9741	0.00 ± 0.06	0.020	0.053
Piped water	Mean (SD)	9741	0.32 ± 0.47	− 0.049	** < 0.001**
Public water	Mean (SD)	9741	0.14 ± 0.34	0.001	0.893
Well water	Mean (SD)	9741	0.27 ± 0.45	0.028	**0.006**
Surface water	Mean (SD)	9741	0.25 ± 0.43	0.024	**0.018**
Other source water	Mean (SD)	9741	0.02 ± 0.13	− 0.004	0.700
No toilet	Mean (SD)	9741	0.02 ± 0.12	0.023	**0.025**
Pit latrine	Mean (SD)	9741	0.74 ± 0.44	0.004	0.678
Flush toilet	Mean (SD)	9741	0.22 ± 0.42	− 0.017	0.086
Other toilet facility	Mean (SD)	9741	0.02 ± 0.15	0.022	**0.031**
Cooking method: Firewood	Mean (SD)	9741	0.52 ± 0.50	0.038	** < 0.001**
Cooking method: Charcoal	Mean (SD)	9741	0.14 ± 0.34	− 0.011	0.257
Cooking method: Kerosene stove	Mean (SD)	9741	0.04 ± 0.19	0.022	**0.028**
Cooking method: Gas stove	Mean (SD)	9741	0.27 ± 0.44	− 0.047	** < 0.001**
Cooking method: Electric stove	Mean (SD)	9741	0.03 ± 0.16	0.003	0.753
Cooking method: Other	Mean (SD)	9741	0.01 ± 0.11	0.013	0.202

Mean scores on Binary Response scale (0 = No and 1 = Yes); *p-value* significance level, *SD* Standard deviation.

Significant values are in bold.

### Association between stress and psychiatric disorders

These are summarized in Table 4. There were significant positive correlations between stress and all PDSQ psychiatric disorders (*p* < 0.001) which ranged from 0.438 for depression (highest) to 0.211 for drug abuse/dependence (lowest).

**Table 4:** Correlation between total stress score and psychiatric disorder scores.

Pearson correlation	1	2	3	4	5	6	7	8
Total WERC Stress Score:	1							
WERCAP-Bipolar Disorder Score	0.415**	1						
WERCAP-Schizophrenia Score	0.418**	0.660**	1					
Major Depressive Disorder Score	0.438**	0.504**	0.487**	1				
PTSD Score	0.362**	0.348**	0.382**	0.514**	1			
Bulimia/Binge Eating Disorder Score	0.308**	0.302**	0.353**	0.488**	0.412**	1		
Obsessive Compulsive Disorder Score	0.311**	0.322**	0.353**	0.426**	0.391**	0.377**	1	
Panic Disorder Score	0.339**	0.338**	0.393**	0.497**	0.449**	0.442**	0.526**	1
Psychosis Score	0.299**	0.329**	0.445**	0.467**	0.443**	0.466**	0.476**	0.563**
Agoraphobia Score	0.300**	0.303**	0.339**	0.432**	0.389**	0.396**	0.461**	0.502**
Social Phobia Score	0.311**	0.334**	0.327**	0.462**	0.376**	0.363**	0.469**	0.453**
Alcohol Abuse/Dependence Score	0.217**	0.183**	0.242**	0.316**	0.269**	0.380**	0.211**	0.298**
Drug Abuse/Dependence Score	0.211**	0.178**	0.231**	0.295**	0.267**	0.371**	0.199**	0.258**
Generalized Anxiety Disorder Score	0.345**	0.409**	0.386**	0.519**	0.413**	0.376**	0.425**	0.486**
Somatization Disorder Score	0.275**	0.294**	0.291**	0.411**	0.333**	0.344**	0.301**	0.391**
Hypochondriasis Score	0.280**	0.276**	0.316**	0.397**	0.339**	0.350**	0.304**	0.424**

Pearson correlation	9	10	11	12	13	14	15	16
Total WERC Stress Score:								
WERCAP-Bipolar Disorder Score								
WERCAP-Schizophrenia Score								
Major Depressive Disorder Score								
PTSD Score								
Bulimia/Binge Eating Disorder Score								
Obsessive Compulsive Disorder Score								
Panic Disorder Score								
Psychosis Score	1							
Agoraphobia Score	0.472**	1						
Social Phobia Score	0.425**	0.573**	1					
Alcohol Abuse/Dependence Score	0.334**	0.284**	0.275**	1				
Drug Abuse/Dependence Score	0.312**	0.247**	0.233**	0.617**	1			
Generalized Anxiety Disorder Score	0.424**	0.468**	0.556**	0.275**	0.276**	1		
Somatization Disorder Score	0.354**	0.377**	0.377**	0.298**	0.290**	0.424**	1	
Hypochondriasis Score	0.383**	0.376**	0.365**	0.305**	0.314**	0.451**	0.525**	1

**Correlation is significant at the 0.01 level (2-tailed).

### Independent predictors of stress

Table 5 summarizes the independent predictors of stress. Socio-demographic predictors of stress were female gender, 18–20 year old age group and having 4 and above siblings. Well source of water was the only economic significant predictor of stress. All psychiatric conditions were predictors of stress (*p* < 0.05) except two i.e. agoraphobia and somatization disorders.

**Table 5:** Independent predictors of stress (mean scores of economic indicators and absolute scores of psychiatric conditions).

Variable	Category	Beta	SE	95% CI		*p*-value
				Lower	Upper	
Gender	Male	Ref				
	Female	1.13	0.50	0.16	2.10	**0.022**
Age (years)	15–17	Ref				
	18–20	− 3.13	1.18	− 5.45	− 0.82	**0.008**
	21–24	− 2.25	1.18	− 4.56	0.06	0.057
	≥ 25	− 1.50	1.53	− 4.49	1.49	0.327
Religion	Protestant	− 2.03	1.25	− 4.47	0.42	0.105
	Catholic	− 1.27	1.27	− 3.77	1.22	0.318
	Muslim	0.80	1.68	− 2.50	4.09	0.636
	Other	Ref				
Number of siblings	0–3	Ref				
	≥ 4	1.41	0.50	0.44	2.38	**0.005**
Level of education	High School	0.51	0.78	− 1.01	2.04	0.510
	College	1.21	0.66	− 0.08	2.50	0.067
	University	Ref				
Electricity	Mean (SD)	0.05	0.65	− 1.22	1.31	0.944
Television	Mean (SD)	− 0.77	0.63	− 2.00	0.45	0.216
Earth floor	Mean (SD)	0.72	0.85	− 0.94	2.38	0.394
Cement floor	Mean (SD)	− 1.23	0.63	− 2.46	0.01	0.051
Piped water	Mean (SD)	0.33	0.75	− 1.14	1.80	0.661
Well water	Mean (SD)	1.77	0.75	0.30	3.23	**0.018**
Surface water	Mean (SD)	0.77	0.77	− 0.74	2.29	0.318
No toilet	Mean (SD)	− 2.00	1.96	− 5.83	1.84	0.308
Other toilet facility	Mean (SD)	1.20	1.58	− 1.89	4.29	0.447
Cooking method: Firewood	Mean (SD)	0.49	0.70	− 0.87	1.85	0.481
Cooking method: Kerosene stove	Mean (SD)	1.44	1.37	− 1.25	4.12	0.294
Cooking method: Gas stove	Mean (SD)	− 0.74	0.75	− 2.20	0.73	0.324
WERCAP-Bipolar Disorder Score		0.49	0.04	0.41	0.56	** < 0.001**
WERCAP-Schizophrenia Score		0.38	0.03	0.32	0.45	** < 0.001**
Major Depressive Disorder Score		0.90	0.08	0.75	1.05	** < 0.001**
PTSD Score		0.68	0.08	0.53	0.84	** < 0.001**
Bulimia/Binge Eating Disorder Score		0.43	0.15	0.14	0.72	**0.003**
Obsessive Compulsive Disorder Score		0.60	0.14	0.33	0.86	** < 0.001**
Panic Disorder Score		0.40	0.15	0.12	0.69	**0.005**
Psychosis Score		− 0.87	0.21	− 1.28	− 0.47	** < 0.001**
Agoraphobia Score		0.12	0.11	− 0.08	0.33	0.249
Social Phobia Score		0.23	0.08	0.08	0.38	**0.003**
Alcohol Abuse/Dependence Score		0.53	0.25	0.04	1.02	**0.035**
Drug Abuse/Dependence Score		0.58	0.27	0.05	1.10	**0.031**
Generalized Anxiety Disorder Score		0.25	0.11	0.05	0.46	**0.016**
Somatization Disorder Score		0.40	0.24	− 0.07	0.87	0.099
Hypochondriasis Score		0.53	0.23	0.07	0.98	**0.022**

*SE* Standard Error, *CI* Confidence Interval, *p-value* significance level.

Significant values are in bold.

## Discussion

### The strength of the study

We present the findings of a study that has several strengths: A large sample, concurrent and uniform collection of data through three strata of students in Kenya (high school, college and university). In addition to stressors, we collected three key sets of data for comparison: Socio-demographic, economic indicators and psychiatric disorders. This concurrent collection of data places the stressors in a context that contributes to informed context-appropriate intervention. This is the first time as far as we can establish from the literature that such a study is being reported in Kenya.

### The response rate

The high response rate reported in this study is not unique and has been reported in our multiple studies^46,47^. There are several explanations for this. In Kenya, there is a tendency to take seriously and participate in activities including research activities that touch on education. This is because of the traditional importance attached to education as the most viable and accepted way to succeed in life. Any activity that is perceived to have the potential to improve academic performance through better mental health is warmly embraced. This acceptance cuts across the whole community ranging from the students, parents, teachers and the community members. In addition to this we undertook comprehensive multi stakeholder’s engagement to explain the nature of the study. Another reason for the high response rate is the method we used in administering the questionnaires in a classroom situation and the freedom to move at their own individual rate. We further requested the students to go back to the questions to see if they had missed any question that they felt free to answer.

### Socio-demographic and wealth index

The socio-demographic data found in this study can be understood in context. The male overrepresentation does not reflect the general population trends but the manner in which the sample was obtained. The high school students were recruited when the schools were closed and only those who could make it to the designated data collection points were included in the study. These were likely to be boys who have greater freedom than girls to be allowed to go to those collection points on their own. Secondly, most of the colleges were technical and therefore likely to attract boys than girls. The high mean age of 21.4 (range 15–43) reflects the fact that colleges and university students were the majority (usually 18 + as compared to high school (14–17)). However, late age enrollment in schools or even college/university levels explains the wide age range. Further, unlike high school students who were recruited away from schools, college and university students were recruited at their institution, and therefore a large number of students were easily available. Being a student population, it is not surprising that the majority were not married. The majority of protestant religion is a reflection of the national trends^48^. That the majority of students were 1st or 2nd born is a reflection of the increasing trends for smaller families in Kenya^49^. The almost equal distribution of the wealth index is a reflection of equal opportunities to go to school (a legal requirement, college and university based on academic performance and government scholarships). However, those parents who can afford have the option to send their children to private and more expensive institutions. This may be the explanation for the lower prevalence of the wealth index quintile 5, given that we studied public institutions. Though we found extreme indicators of poverty associated with stress, it was quintile 2, not quintile 1 that was associated with stress. We attribute this to the influence of electricity and television, an indicator of the high wealth quintile that caused quintile 2 to be more associated with stress. The roles of electricity and television as causes of stress are explained in more detail in the following sub-heading.

### The prevalence and severity of different stressors

The overwhelming majority of the students scored negative on all the various stressors ranging from 30 to 90% for the different stressors but on average 70% of all the students did not experience any form of stress at the time of the study. This is as compared to 95.9% of students in an Ethiopian study referenced in the introduction which found a stress prevalence of only 4.1% among college students. Thus, our students were more stressed than was found in the Ethiopian study. However, this could be a reflection of the methodologies in the two studies, more particularly on the categorization of levels of stress in the Kenyan study. The decreasing prevalence in severity (from little to severe) from an average of 20%, 10%, 5%, and less than 5% that emerges in this study suggests the need to categorize levels of stress and then prioritize on those participants who are the most severely affected, given the scarce resources available to institutions. This calls for screeners that categorize levels of stress administered to students attending health facilities at individual level or to group screens to reach large numbers of students not otherwise attending health facilities. The WERC Stress which has been validated in Kenya comes in handy.

The emerging trends of different types of stressors also provide fertile ground for informed intervention. Money and finances are to some extent a reflection of the family's economic backgrounds on how much they can be supportive of their children as also was found in a China study as referenced in the introduction.. Institution-based supportive and counseling services may not do much about economic factors operating in the environment of the families and over which the student may have little control. However, the supportive and counselling services available at the institutions should be aware of these factors and at the least bring them up in their services and talk about them, but never ignore them as not relevant. This may be the only time the students ever have on ventilating on any economic difficulties that may be adversely affecting them. This approach would provide supportive and cognitive behavioral therapy to enable the students to cope best with their financial status. This finding of finance as an issue amongst students in our study is not unique and has been found in other studies referenced in the introduction. The same approach is relevant when dealing with death (the 2nd most important stressor). It is to be noted that family related issues in different forms were some of the leading stressors. Therefore, family members should be involved to the extent possible. This is more realistic with increasing use of technology for distant and virtual consultations. Our finding that alcohol or drug use was the least stressor is similar to a finding in America by one of the authors of this paper and referenced in the introduction. . It is possible that these are used as coping mechanisms and as self-medication to ameliorate the level of perceived stress. Therefore, substance abuse should always be raised with the students during intervention sessions for two reasons—to promote positive coping and to prevent dependency.

### Association between stress score and socio-demographic and economic indicators

We found that the female gender was associated with an increased level of stress, a finding similarly found in multiple studies as referenced in the introduction. This could imply that females face more stressors in daily life than males and/or they may tend to internalize stress and stress coping more than the male gender as found in a study by^28^ referenced in the introduction. However, the possibility that male students do not internalize stress does not necessarily mean that they do not deal with stress in other ways such as externalizing disruptive behavior. Whichever way of dealing with stress in either males or females should be explored as part of the intervention. Religion may have a protective role in relation to stress through a belief in a higher controlling power, hence more stress in those who do not profess any religion. It is therefore important not to belittle the role of religion from the point of view of the students during intervention. The finding that university students had less stress levels compared particularly with college students, closely followed by high school students, is understandable in the local context. High school students have the stress of uncertainty of the future, compared with university students who are more assured, given a much higher competitive advantage in jobs and other opportunities. Most high school students do not make it to college, and most college students do not make it to the university, thus the different mean stress levels in the three education groups may reflect differences in their confidence about the future. It is also possible that students at these three levels are not the same even if the opportunity was not the most important consideration.

A surprising finding in our study was that electricity and television as household items were associated with reduced stress. Electricity is necessary for television. We would have expected that television would have increased stress level as reported in our introduction since television exposes children to social media and different lifestyles that the children aspire for even if not context-appropriate or affordable. This could put the children on a collision course with the parents and therefore a source of stress for the children. We speculate that the decrease of stress associated with television in our findings is due to the fact that availability of electricity and television is in most cases associated with better family wealth status in the Kenyan context and so is a motor vehicle, which was not associated with stress. Despite the enhanced rural electrification program that makes electricity available at much subsidized cost and also the increased solar energy availability, they are still relatively more available to those who can afford them. Availability or non-availability of electricity is an economic and environmental factor at home and therefore outside the control of the student. However, like other factors outside the control of the student, it does not preclude any discussion on this and how it affects the student. Earth floor for the family house, well-water for the family house, surface-water for the family house, no toilet or kerosene stove followed by firewood use as cooking method by the family are indicators of poor economic status. We therefore conclude that poverty at the home level has something to contribute to stress. This poverty explains the noted money and financial matters as the leading source of stress in the students. The explanation for the “well source of water” for the household as an independent predictor of stress can be deduced from the overall impact of economic status and how to be aware of this in the course of intervention. Our study found age (18–20 year old) to be a predictor of stress a finding consistent to that in Nigeria. This is not surprising as this is the age group at which young people do national exams to determine their future career either not to go on with education and start looking for income or qualify to go to college or qualify to the more preferred choice of university. It is also the time the young people transit through these various options e.g. looking for a job or being in their first year of studies in the tertiary levels away from their protective homes. There is little intervention can do about national exams or on the transition but they can be brought up for discussion during the interventions with a view to promoting positive coping mechanisms. It is not surprising that having 4 siblings and above was a predictor of stress in our study. Having many siblings could expose one to limited/lack of basic needs as parents struggle to care for the needs of all the children. This coupled with the divided attention one receives from the parents as compared to the undivided attention one would receive if there were no siblings or fewer siblings could contribute to the stress of the student. These could still be brought up during the interventions in order to promote more positive coping mechanisms.

### Stress and psychiatric disorders

The widespread association between stress and psychiatric disorders found in our study has been reported in various other studies reported in the introduction. This can be explained in several ways: Firstly, the psychiatric disorder could play a causative or catalytic role in the precipitation of stress. The reverse is also possible with stress playing a similar role in relation to psychiatric disorders. Thirdly, it could be that these are chance associations. Whatever the nature of the relationship, these findings suggest routine screening for stress in any students presenting with a psychiatric disorder and also routine screening for psychiatric disorders in any student presenting with stress. This co-occurrence of stress and psychiatric disorders stands even after analyzing for the independent predictor of stress, emphasizing the need for interventions that address these two conditions concurrently.

Thus, we conclude from our study that stress is a multi-factored phenomenon with many associated co-occurrences. These multi-factored co-occurrences call for a multi-factored approach to stress intervention and management in high school, college and university students in the Kenyan context.

### Possibilities for interventions

These have already been alluded to in the various sub-headings discussed above and can be summarized as follows;

1. Most of the identified stressors or associated complications are amenable to intervention. An identified mental disorder can be intervened through the usual clinical management using pharmacotherapy or cognitive behavioral therapy (CBT) or both. Those stressors that are independent of the students such as economic factors in the family about which little can be done to change, could still be subjected to intervention through CBT so as to provide better coping mechanism and/or enhanced resilience.
2. Awareness is a key intervention with the potential to prevent complications arising out of the stressors and/or to minimize the complications of the stressors by better coping skills. Any interventions such as public and individual psychoeducation is a critical first stage intervention to create awareness. However, we recommend prioritization of a public health approach so as to reach critical numbers including those not yet experiencing the stressors or already experience the stressors but not quite aware of what is going on in themselves. Enhanced awareness of the various stressors whether by the students, institutions, the family is a first critical stage to motivated screening and health seeking behavior by the students. Such awareness at the level of the institution provides basis for enhanced services to meet the increased demand for screening by the students through provision of appropriate expertise and appropriate intervention. In a country where there is a dearth of mental health specialists, task shifting to trained and supervised non-specialist mental health workers comes very handy. Further, psychoeducation as an intervention provides the basis for the families to engage with their students towards health seeking behavior.

We have thus achieved the four aims of our study summarized at the end of the Introduction.

## Limitations

We did not have a qualitative component in the study. We were therefore not able to tease out the nuances that would have explained the perceived stressors. Secondly, we cannot extrapolate from our findings to other institutions and therefore the need as indicated under the possibilities, for each institution to do their screening for context-appropriate interventions. At best, our study has demonstrated the feasibility of undertaking this kind of screening.

## Data Availability

All data presented in this review are available upon request from the corresponding author, using our laid down data sharing policy.

## References

[1] Braceland FJ (1976). The stress of life. Psychiatr. Ann..

[2] Lazarus RS, Folkman S (1984). Stress, Appraisal, and Coping.

[3] Bamuhair SS, Al Farhan AI, Althubaiti A, Agha S, ur Rahman S, Ibrahim NO (2015). Sources of stress and coping strategies among undergraduate medical students enrolled in a problem-based learning curriculum. J. Biomed. Educ..

[4] Hamaideh SH (2011). Stressors and reactions to stressors among university students. Int. J. Soc. Psychiatry.

[5] Banerjee N, Chatterjee I (2016). Academic stress, suicidal ideation & mental wellbeing among 1st semester & 3rd semester medical, engineering & general stream students. Res. World: J. Arts Sci. Commer..

[6] Buchanan JL (2012). Prevention of depression in the college student population: A review of the literature. Arch. Psychiatr. Nurs..

[7] Yikealo D, Yemane B, Karvinen I (2018). The level of academic and environmental stress among college students: A case in the college of education. Open J. Soc. Sci..

[8] Pariat ML, Rynjah MA, Joplin M, Kharjana MG (2014). stress levels of college students: Interrelationship between stressors and coping strategies. IOSR J. Humanit. Soc. Sci..

[9] Ramón-Arbués E, Gea-Caballero V, Granada-López JM, Juárez-Vela R, Pellicer-García B, Antón-Solanas I (2020). The prevalence of depression, anxiety and stress and their associated factors in college students. Int. J. Environ. Res. Public Health.

[10] Zeng Y, Wang G, Xie C, Hu X, Reinhardt JD (2019). Prevalence and correlates of depression, anxiety and symptoms of stress in vocational college nursing students from Sichuan, China: A cross-sectional study. Psychol. Health Med..

[11] Abebe AM, Kebede YG, Mengistu F (2018). Prevalence of stress and associated factors among regular students at Debre Birhan governmental and nongovernmental health science colleges North Showa Zone, Amhara Region, Ethiopia 2016. Psychiatry J..

[12] Borjalilu S, Mohammadi A, Mojtahedzadeh R (2015). Sources and severity of perceived stress among Iranian medical students. Iran. Red Crescent Med. J..

[13] Oketch-Oboth JW, Okunya LO (2018). The relationship between levels of stress and academic performance among University of Nairobi students. Int. J. Learn. Dev..

[14] Shah SSNH, Laving A, Okech-Helu VC, Kumar M (2021). Depression and its associated factors: Perceived stress, social support, substance use and related sociodemographic risk factors in medical school residents in Nairobi, Kenya. BMC Psychiatry.

[15] Aiyegbusi AI, Akinbo SR, Adebisi OB (2022). Patterns and the relationship between socio-demographic variables and perceived stress among undergraduate students of the College of Medicine, University of Lagos, Nigeria. Univ. Lagos J. Basic Med. Sci..

[16] McCann CM, Beddoe E, McCormick K, Huggard P, Kedge S, Adamson C, Huggard J (2013). Resilience in the health professions: A review of recent literature. Int. J. Wellbeing.

[17] Muna S, Atinkut Z (2018). Prevalence and associated factors of stress among undergraduate students in Ambo University: Implication for Intervention. Int. J. Psychol. Couns..

[18] Ong B, Cheong KC (2009). Sources of stress among college students—The case of a credit transfer program. Coll. Stud. J..

[19] Robotham D (2008). Stress among higher education students: Towards a research agenda. High. Educ..

[20] Arnsten AFT (2009). Stress signalling pathways that impair prefrontal cortex structure and function. Nat. Rev. Neurosci..

[21] Britt SL, Mendiola MR, Schink GH, Tibbetts RH, Jones SH (2016). Financial stress, coping strategy, and academic achievement of college students. J. Financ. Couns. Plan..

[22] Kwaah CY, Essilfie G (2017). Stress and coping strategies among distance education students at the University of Cape Coast, Ghana. Turk. Online J. Distance Educ..

[23] Tesfaw AA, Yitayih TT (2018). A study on financial stress and coping strategies among students in Rift Valley University, Ethiopia National Disaster Risk Management Commission, Ethiopia 2. Hum. Soc. Sci. J..

[24] Wilks, S. E. Resilience amid academic stress: The moderating impact of social support among social work students. *Adv. Soc. Work* **9**(2), 106–125. https://doi.org/10.18060/51 (2008).

[25] Jain A, Verma S (2016). Prevalence of stress and coping strategies among college students. J. Adv. Med. Dent. Sci. Res..

[26] Madhyastha S, Latha KS, Kamath A (2014). Stress, coping and gender differences in third year medical students. J. Health Manag..

[27] Ramachandran V, Chandrasekar K, Nanjudan P, Mani M, Baskaran S (2017). Evaluation of stress among college students. World J. Pharm. Med. Res..

[28] Achenbach TM, Ndetei DM (2012). A.3—Clinical models for child and adolescent behavioral, emotional and social problems. IACAPAP Textb. Child Adolesc. Mental Health.

[29] Alharbi E, Smith A (2018). A review of the literature on stress and wellbeing among international students in English-speaking countries. Int. Educ. Stud..

[30] Gao W, Ping S, Liu X (2020). Gender differences in depression, anxiety, and stress among college students: A longitudinal study from China. J. Affect. Disord..

[31] Oseyomon P (2015). Students’ attributes and level of stress in the University of Benin. Esut J. Account..

[32] Yang T, Yang XY, Yu L, Cottrell RR, Jiang S (2017). Individual and regional association between socioeconomic status and uncertainty stress, and life stress: A representative nationwide study of China. Int. J. Equity Health.

[33] Ndetei DM, Mutiso VN, Weisz JR, Okoth CA, Musyimi C, Muia EN, Osborn TL, Sourander A, Wasserman D, Mamah D (2022). Socio-demographic, economic and mental health problems were risk factors for suicidal ideation among Kenyan students aged 15 plus. J. Affect. Disord..

[34] Jung SJ, Winning A, Roberts AL, Nishimi K, Chen Q, Gilsanz P, Sumner JA, Fernandez CA, Rimm EB, Kubzansky LD (2019). Posttraumatic stress disorder symptoms and television viewing patterns in the Nurses’ Health Study II: A longitudinal analysis. PLoS ONE.

[35] Bangasser DA, Valentino RJ (2014). Sex differences in stress-related psychiatric disorders: Neurobiological perspectives. Front. Neuroendocrinol..

[36] Mamah D, Owoso A, Sheffield JM, Bayer C (2014). The WERCAP Screen and the WERC Stress Screen: Psychometrics of self-rated instruments for assessing bipolar and psychotic disorder risk and perceived stress burden. Compr. Psychiatry.

[37] Mumford DB, Minhas FA, Akhtar I, Akhter S, Mubbashar MH (2000). Stress and psychiatric disorder in urban Rawalpindi: Community survey. Br. J. Psychiatry.

[38] Graham JE, Christian LM, Kiecolt-Glaser JK (2006). Stress, age, and immune function: Toward a lifespan approach. J. Behav. Med..

[39] Cherkil S, Gardens SJ, Soman DK (2013). Coping styles and its association with sources of stress in undergraduate medical students. Indian J. Psychol. Med..

[40] Pascoe MC, Hetrick SE, Parker AG (2020). The impact of stress on students in secondary school and higher education. Int. J. Adolesc. Youth.

[41] Zimmerman M, Mattia JI (2001). The Psychiatric Diagnostic Screening Questionnaire: Development, reliability and validity. Compr. Psychiatry.

[42] Campbell DT, Fiske DW (1959). Convergent and discriminant validation by the multitrait-multimethod matrix. Psychol. Bull..

[43] Ndetei D, Pike K, Mutiso V, Tele A, Gitonga I, Rebello T, Musyimi C, Mamah D (2019). The psychometric properties of the Washington Early Recognition Center Affectivity and Psychosis (WERCAP) screen in adults in the Kenyan context: Towards combined large scale community screening for affectivity and psychosis. Psychiatry Res..

[44] Hsieh CJ, Godwin D, Mamah D (2016). Utility of Washington early recognition center self-report screening questionnaires in the assessment of patients with schizophrenia and bipolar disorder. Front. Psychiatry.

[45] Smits J, Steendijk R (2015). The International Wealth Index (IWI). Soc. Indic. Res..

[46] Ndetei DM, Khasakhala LI, Mutiso V, Ongecha-Owuor FA, Kokonya DA (2009). Patterns of drug abuse in public secondary schools in Kenya. Subst. Abuse.

[47] Ndetei DM, Khasakhala LI, Mutiso V, Ongecha-Owuor FA, Kokonya DA (2010). Drug use in a rural secondary school in Kenya. Subst. Abuse.

[48] KNBS. *2019 Kenya Population and Housing Census Volume IV: Distribution of Population by Socio-Economic Characteristics* (2019).

[49] Kenya National Bureau of Statistics (KNBS). *2019 Kenya Population and Housing Census Volume III: Distribution of Population by Age and Sex* (2019).

